# Аn Unusual Case of an Epidural Lesion of the Lumbar Spine: Tumor or Hematoma?

**DOI:** 10.7759/cureus.50256

**Published:** 2023-12-10

**Authors:** Plamen Penchev, Petar-Preslav Petrov, Vladislav Velchev, Ilko Ilyov, Edvin Vasvi

**Affiliations:** 1 Department of Medicine, Medical University of Plovdiv, Plovdiv, BGR; 2 Department of Anatomy, Histology and Embriology, Medical University of Plovdiv, Plovdiv, BGR; 3 Neurological Surgery, Saint Anna Hospital, Varna, BGR

**Keywords:** lumbar spine, mri, epidural tumor, case report, acute epidural hematoma

## Abstract

Spinal epidural hematoma is a rare clinical entity with an incidence of approximately one per 1,000,000 patients per year. Spinal epidural hematoma is a lesion that can cause spinal cord compression or cauda equina syndrome. We report a clinical case of а 69-year-old male patient who presented to the Neurosurgery Clinic of the General Hospital for Active Treatment “Dobrich” with pain and weakness in both legs for two months after falling in the bathroom. MRI revealed an L2-L3 fracture and a formation in the L2-L3 epidural space, which was compressing the nerve roots. An operative treatment was performed under general anesthesia and, intraoperatively, it was discovered that the formation was a hematoma. Aspiration of the hematoma and decompression of the spinal canal were performed. An L2-L3 stabilization with pedicle screws was done due to total laminectomy and potential instability. Postoperatively, the patient was mobilized on the day after intervention, and no surgery-related complications were observed. The patient experienced relief from his symptoms and was discharged on the fifth day. Six months post-surgery, the patient started to experience pain in his left leg. Radiography showed coxarthrosis on the left hip joint and the patient was referred to the orthopedics for further treatment.

## Introduction

Spinal epidural hematoma is an uncommon acute disorder that can develop on its own or as a result of trauma. About one out of every one million patients experiences a spinal epidural hematoma each year [1]. It is a lesion that may result in the compression of the spinal cord and/or cauda equina [2]. Due to their enhanced bulk, chronic spinal epidural hematomas, however, can pass for spinal tumors [2,3]. The possible risk factors include anticoagulants, disc herniation, hypertension, uremia, chronic kidney disease, dengue fever, coagulopathy, and coronavirus disease 2019 (COVID-19) [4]. MRI can provide the basis for the diagnosis with its particular morphological features and severity. In this study, we emphasize that chronic spinal hematoma should be considered in the differential diagnosis of similar scenarios and for surgical planning. Differential diagnosis of lesions in the lumbar spine can be challenging since prompt management is critical for optimal outcomes. The diagnosis of hematoma is of paramount importance, especially in cases with neurological deterioration. Spinal epidural hematoma is a rare condition, and this case highlights its occurrence post-trauma, emphasizing its uncommon nature as it can mimic other spinal conditions such as tumors. We lay stress on the importance of detailed imaging and differential diagnosis. We undertake a comprehensive case study, addressing the diagnostic, therapeutic, and follow-up aspects of spinal epidural hematoma, and offer insights into its management.

This article was previously presented as a meeting abstract at the XIV National Conference of Rare Diseases and Orphan Drugs in Plovdiv, Bulgaria, on September 29, 2023.

## Case presentation

**Figure 1:**
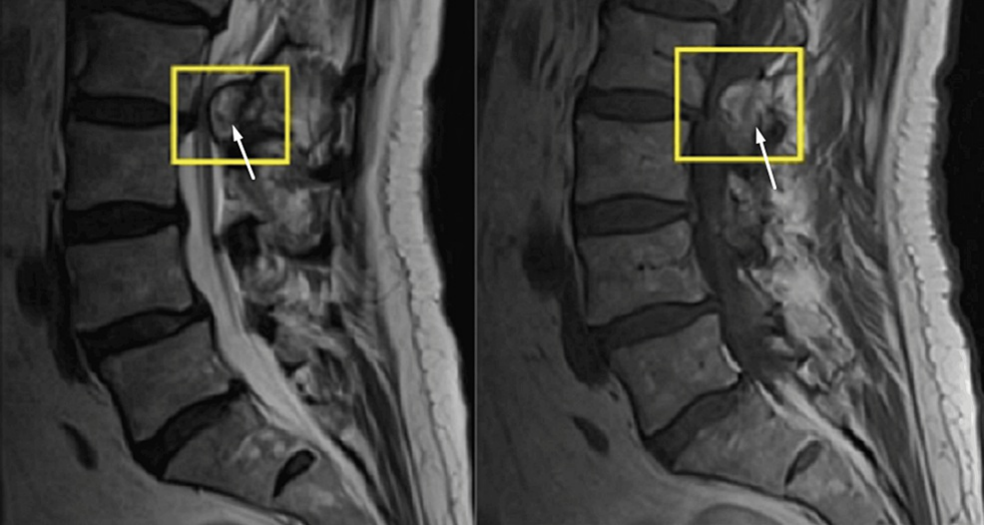
Preoperative MRI: sagittal plane The MRI found evidence of a formation in the L2-L3 epidural space compressing the nerve structures MRI: magnetic resonance imaging

We discuss a clinical case of a 69-year-old male patient who presented to the Neurosurgery Clinic of the General Hospital for Active Treatment “Dobrich” with complaints of pain and weakness in both legs for two months after falling in the bathroom. MRI revealed an L2-L3 fracture and a formation in the L2-L3 epidural space that was compressing the nerve structures (Figure 1). We analyzed the STIR images, but they showed only the tumor lesion, with no other abnormality. The patient did not require contrast-enhanced MRI because the native MRI showed a lesion with significant compression of the dural sac resulting in a neurological deficit. Since no contrast was added in the first MRI so that surgical treatment to decompress the neural structures could be promptly initiated, no further imaging was deemed necessary and the patient underwent operative treatment soon after the diagnosis.

**Figure 2:**
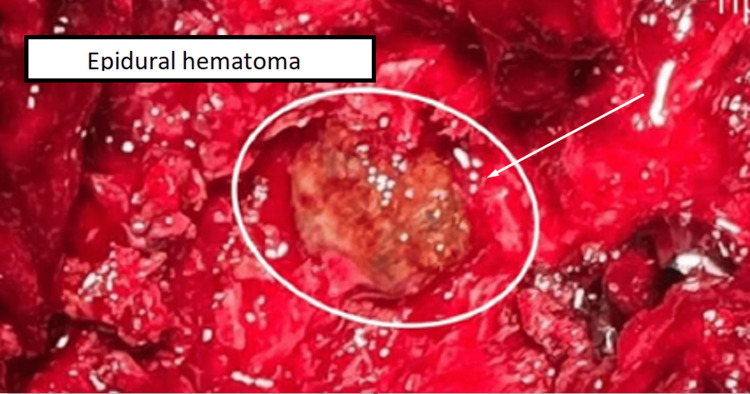
Intraoperative photography - 1 The image shows intraoperative evidence of an epidural hematoma compressing the spinal cord

**Figure 3:**
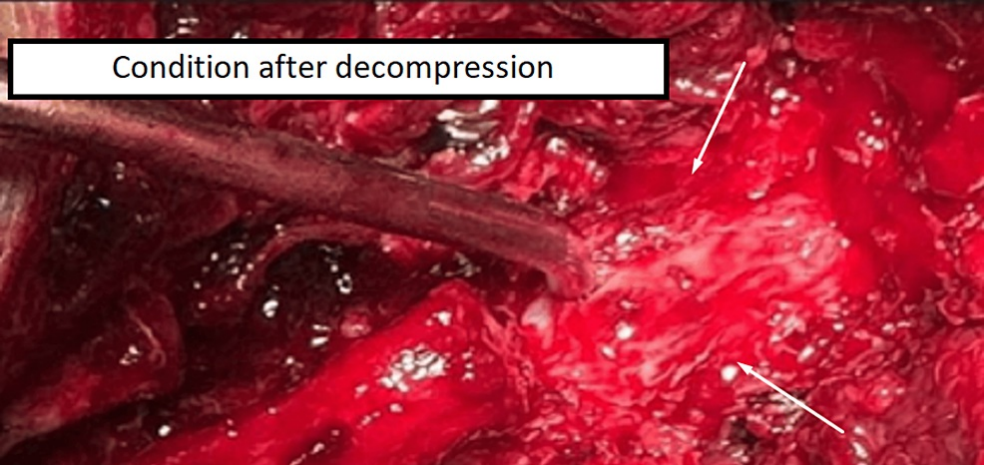
Intraoperative photography - 2 The image shows the condition after the aspiration of the hematoma and decompression of the spinal cord

**Figure 4:**
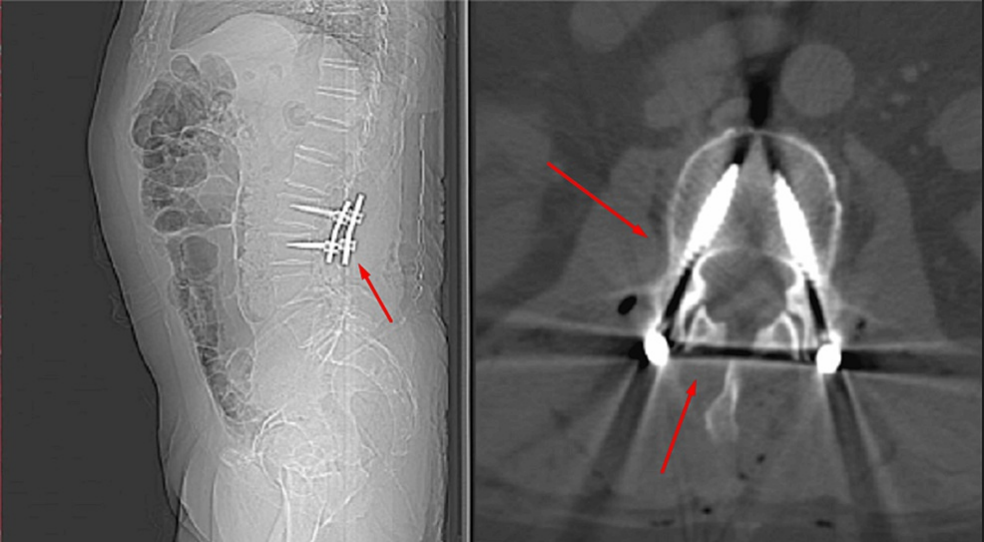
Postoperative radiography Radiography showed evidence of a successful stabilization of L2-L3 level with peduncle screws. No evidence of complications or pathological instability was seen

The patient underwent operative treatment under general anesthesia and, intraoperatively, it was discovered that the formation was a hematoma with a capsule and blood content (Figure 2). Aspiration of the hematoma and decompression of the spinal canal were performed (Figure 3). A total laminectomy of the L2-L3 level was also done. Spinal arthrodesis was performed due to total laminectomy and partial facetectomy, and to avoid iatrogenic instability (Figure 4).

**Figure 5:**
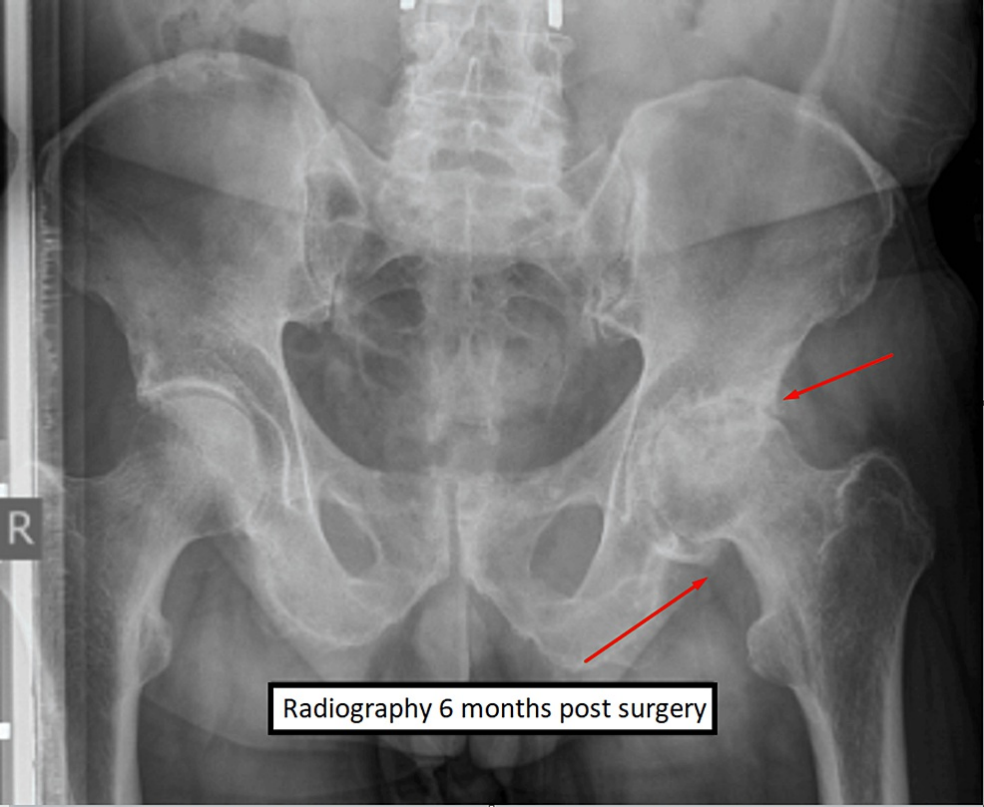
Radiography six months post-surgery Radiography showed evidence of coxarthrosis of the left hip joint

Postoperatively, the patient was verticalized on the day after intervention., and no surgery-related complications were observed. The patient experienced relief from his symptoms and was discharged on the fifth day. In the preoperative period, the patient visited the operator's (surgeon's) office on crutches. Postoperatively, after the decompression of the nerve structures and rehabilitation, the patient moved without crutches and showed significant neurological improvement and recovery. A partial neurological deficit persists, but it does not significantly impair the quality of life and the patient can perform daily activities without any aid in his movement. Extensive decompression of the neural structures led to good recovery and improvement in neurological status. Six months after the surgery, the patient complained of pain in his left leg. Radiography revealed coxarthrosis on the left hip joint and the patient was referred to the orthopedist for further treatment (Figure 5).

## Discussion

Roughly one out of every one million patients experiences a spinal epidural hematoma annually, which accounts for less than 1.7% of all spinal traumas [1] Epidural spinal hematomas can develop following trauma or on their own. Epidural anesthesia, lumbar punctures, falls, fractures, and hemorrhaging following surgery are examples of traumatizing causes [2]. Spinal fractures are seldom associated with traumatic epidural hematomas, even though they might happen in 0.5-7.5% of instances. They are most commonly observed in the dorsal epidural area of the cervical spine [2]. Bleeding from the ruptured valveless venous plexus in the epidural space is the most frequent cause of an epidural hematoma in the spine [2,4,5]. MRI is commonly used to diagnose spinal epidural hematoma. On T1-weighted images, chronic epidural hematomas are often isointense in relation to the spinal cord while they are hyperintense on T2-weighted images. The various enhanced zones seen in hematomas on T2-weighted imaging can be attributed to the hemoglobin degradation products. According to Chang et al., the hematoma is placed epidurally if the dura is visible on sagittal or axial imaging [3]. Various patterns of contrast enhancement associated with acute spinal epidural hematomas have been reported in the literature.

Three instances with peripheral enhancement and three cases with central enhancement have been reported in recent research [3,6,7]. Differential diagnoses such as extramedullary tumors, schwannoma, lymphoma, metastasis, and vascular malformations are included in the MRI enhancement of spinal epidural hematoma [2,8]. Kirwan et al. have described a case with a mixed kind of enhancement (central and peripheral) [9]. Three examples with a spotty kind of augmentation have been documented in studies published in the literature [2,3,9]. According to Elif et al., conservative management of spinal epidural hematoma is recommended in patients who exhibit coagulopathy, a minor neurological disability, or gradual recovery in the early stages of the condition [2]. In our case, the patient's neurological deficiency worsened over the course of two months, and surgery was recommended given the possibility that a tumor had grown and suppressed the spinal cord. Recent studies in the literature have found that anticoagulation and antiaggregant drugs are associated with chronic spinal epidural hematomas in 25-75% of cases [10-12]. In our case, the patient was taking daily aspirin 75 mg and therefore we found a correlation with the claims of previously cited studies. According to Sourani et al., there is an increased risk of developing a spinal epidural hematoma 18 days after experiencing an acute COVID-19 infection [4].

## Conclusions

Spinal epidural hematomas might exhibit several enhancing patterns, which could lead to a false-positive diagnosis of neoplastic processes. Chronic epidural hematomas should be considered in the differential diagnosis of epidural tumor formations, especially in trauma patients. Surgical intervention provides both symptom alleviation and a definitive histological diagnosis in cases where clinical symptoms fail to improve. We emphasize the importance of detailed imaging and differential diagnosis for an accurate diagnosis and prompt treatment.
